# Subtyping irritable bowel syndrome using cluster analysis: a systematic review

**DOI:** 10.1186/s12859-023-05567-8

**Published:** 2023-12-15

**Authors:** Diana Zarei, Amene Saghazadeh, Nima Rezaei

**Affiliations:** 1 School of Medicine, Iran University of Medical Science, Tehran, Iran; 2 Systematic Review and Meta-Analysis Expert Group (SRMEG), Universal Scientific Education and Research Network (USERN), Tehran, Iran; 3 Research Center for Immunodeficiencies, Children’s Medical Center, Tehran University of Medical Sciences, Dr. Qarib St, Keshavarz Blvd, Tehran, 14194 Iran; 4 Integrated Science Association (ISA), Universal Scientific Education and Research Network (USERN), Tehran, Iran; 5 Department of Immunology and Biology, School of Medicine, Tehran University of Medical Sciences, Tehran, Iran

**Keywords:** Cluster analysis, Heterogeneity, Irritable bowel syndrome, Subgroups, Systematic review

## Abstract

**Background:**

Irritable bowel syndrome (IBS) is a common chronic functional gastrointestinal disorder associated with a wide range of clinical symptoms. Some researchers have used cluster analysis (CA), a group of non-supervised learning methods that identifies homogenous clusters within different entities based on their similarity.

**Objective and methods:**

This literature review aims to identify published articles that apply CA to IBS patients. We searched relevant keywords in PubMed, Embase, Web of Science, and Scopus. We reviewed studies in terms of the selected variables, participants’ characteristics, data collection, methodology, number of clusters, clusters’ profiles, and results.

**Results:**

Among the 14 articles focused on the heterogeneity of IBS, eight of them utilized K-means Cluster Analysis (K-means CA), four employed Hierarchical Cluster Analysis, and only two studies utilized Latent Class Analysis. Seven studies focused on clinical symptoms, while four articles examined anocolorectal functions. Two studies were centered around immunological findings, and only one study explored microbial composition. The number of clusters obtained ranged from two to seven, showing variation across the studies. Males exhibited lower symptom severity and fewer psychological findings. The association between symptom severity and rectal perception suggests that altered rectal perception serves as a biological indicator of IBS. Ultra-slow waves observed in IBS patients are linked to increased activity of the anal sphincter, higher anal pressure, dystonia, and dyschezia.

**Conclusion:**

IBS has different subgroups based on different factors. Most IBS patients have low clinical severity, good QoL, high rectal sensitivity, delayed left colon transit time, increased systemic cytokines, and changes in microbial composition, including increased Firmicutes-associated taxa and depleted Bacteroidetes-related taxa. However, the number of clusters is inconsistent across studies due to the methodological heterogeneity. CA, a valuable non-supervised learning method, is sensitive to hyperparameters like the number of clusters and random initialization of cluster centers. The random nature of these parameters leads to diverse outcomes even with the same algorithm. This has implications for future research and practical applications, necessitating further studies to improve our understanding of IBS and develop personalized treatments.

**Supplementary Information:**

The online version contains supplementary material available at 10.1186/s12859-023-05567-8.

## Introduction

Irritable bowel syndrome (IBS) is a chronic functional gastrointestinal (GI) disorder that manifests with abdominal pain, bloating, and altered bowel habits in the absence of any organic disorder or biological markers [1–3]. IBS predominantly affects women [4]. The global prevalence based on ROME III criteria is 9.2%, whereas, based on the ROME IV version, it is estimated at 3.8% [5]. The burden of IBS is significant: individual patients, their families, society, and health care system are all affected [5]. Patients with IBS frequently report lower quality of life (QoL). Particularly, those in the diarrhea-predominant subgroup have lower income because of their absence from work, and their partner and family are also affected by the burden of the disease because these patients might avoid traveling, socializing, etc.

Diagnosing and treating patients with IBS is challenging because there is no single cause [6]. The following possible causes have been considered: mucosal inflammation, mucosal immune activation, changes in intestinal permeability, alteration in the gut microbiome, and post-infectious changes [7]. According to the last published criteria (ROME IV), IBS has four subtypes [8]. However, almost one-third of patients may experience intermittent symptoms. This intermittency complicates subtyping; patients in the same subgroup may have suffered from different underlying mechanisms [9, 10].

To address heterogeneity in research and analysis, various approaches have been used, including subgroup analysis, stratification, regression modeling, and cluster analysis (CA) [11–14]. CA, in particular, has been valuable in identifying distinct subgroups within datasets. However, it is important to choose the appropriate clustering algorithm to ensure reliable and meaningful results. Researchers should carefully consider the best approach to address heterogeneity and enhance the interpretation of their findings.

As a result, a series of researchers decided to use CA, a group of non-supervised learning methods that classifies entities or objects into different homogenous groups or clusters based on their similarity [15–17]. Many algorithms have been introduced, but some are more frequently used [18]. CA has several benefits; for instance, it improves diagnostic criteria to conclude a more comprehensive and meaningful profile, interprets heterogeneous outcomes, and adjusts treatments [19–21]. CA has been used in hypothesis generation, finding a topography, data exploration, and data reduction [22–24]. CA also has some specific usage; it can identify a group of genes with similar biological functions [25] or identify a group of patients that need targeted interventions [22, 23].

CA has several advantages over other methods. It allows researchers to uncover hidden patterns and structures in complex datasets without making assumptions about data distributions making it a versatile technique [14]. However, it is important to note that CA is sensitive to the initial configuration, and choice of algorithm, which means different results can be obtained [26]. To address this, researchers should carefully select appropriate algorithms and validate the stability of the clusters obtained [27]. Furthermore, it is essential to understand that CA alone does not provide casual relationships or explanations, so, further analysis and interpretation are required. Despite these limitations, CA remains a powerful tool for gaining insights into data structures across various fields.

However, there are challenges with using CA [28]. The sample size is calculated based on the variables included in the analysis and the number of identified clusters [29]. To achieve sufficient power, we need to have a large sample size (greater than 200) and split it into two groups: one for training and one for validation [30]. The results can be reported when the same subgroups are obtained in multiple samples of the target population [31]. This article reviews CA studies in IBS.

## Methods

We conducted the present systematic review based on preferred reporting items for systematic reviews and meta-analysis (PRISMA) guidelines (Additional file 1).

### Search strategy

We searched PubMed, Embase, Scopus, and the Web of Science from initiation until November 03, 2022 for relevant published articles in English without restricting the publication date. We used a combination of the keywords related to irritable bowel and cluster analysis. The Additional file 2 includes the queries used for searching in each database.

### Selection criteria

We included studies on patients with IBS who were over the age of 17 years old and had not any organic GI disorder. Non-English and animal articles were excluded.

### Methods of review

The study selection is a four-step process: identification, screening, eligibility, and inclusion. At first, in the identification step, we gather all search records that were obtained from databases and removed duplicates. Then, we screened search results by title/abstract. In the third step, we assessed the potentially eligible articles by their full text and included them in our systematic review if they met the inclusion criteria.

### Data extraction

We evaluated the methods and results section of each included article. Specifically, we retrieved details on the following items: study design, participants’ characteristics, diagnostic criteria, the variables considered for clustering, data collection methods, data preprocessing techniques, clustering algorithms, validation, interpretation of the results, number of clusters, findings, limitations, and suggestions for future studies.

#### Study design

Studies were eligible for inclusion in the present review if their results were obtained from original research. Review articles, systematic reviews, and meta-analyses were excluded. Cohort studies, cross-sectional studies, and case-control studies were included.

#### Participants’ characteristics

We included studies that were conducted on IBS patients, adult participants, and evaluated both sexes.

#### Variables

Selecting relevant variables for discriminating clusters is very important. The variables included were related to GI symptoms, bowel habits, pain, bloating, psychological disturbances, QoL, anorectal function, colon transit time (CTT), anal pressure waves, cytokines levels, mast cell (MC) numbers, and microbial composition.

#### Data collection method

The methods of collecting participants’ data or tools for evaluating patients were reviewed: questionnaire, direct interview, data collection on consecutive days, a rectal examination tool, etc.

#### Data preprocessing methods

Considering that the data obtained from the studies might be different in terms of units or other items, we examined studies to control if they applied standardization and data normalization methods before CA.

#### Cluster analysis

**Fig. 1:**
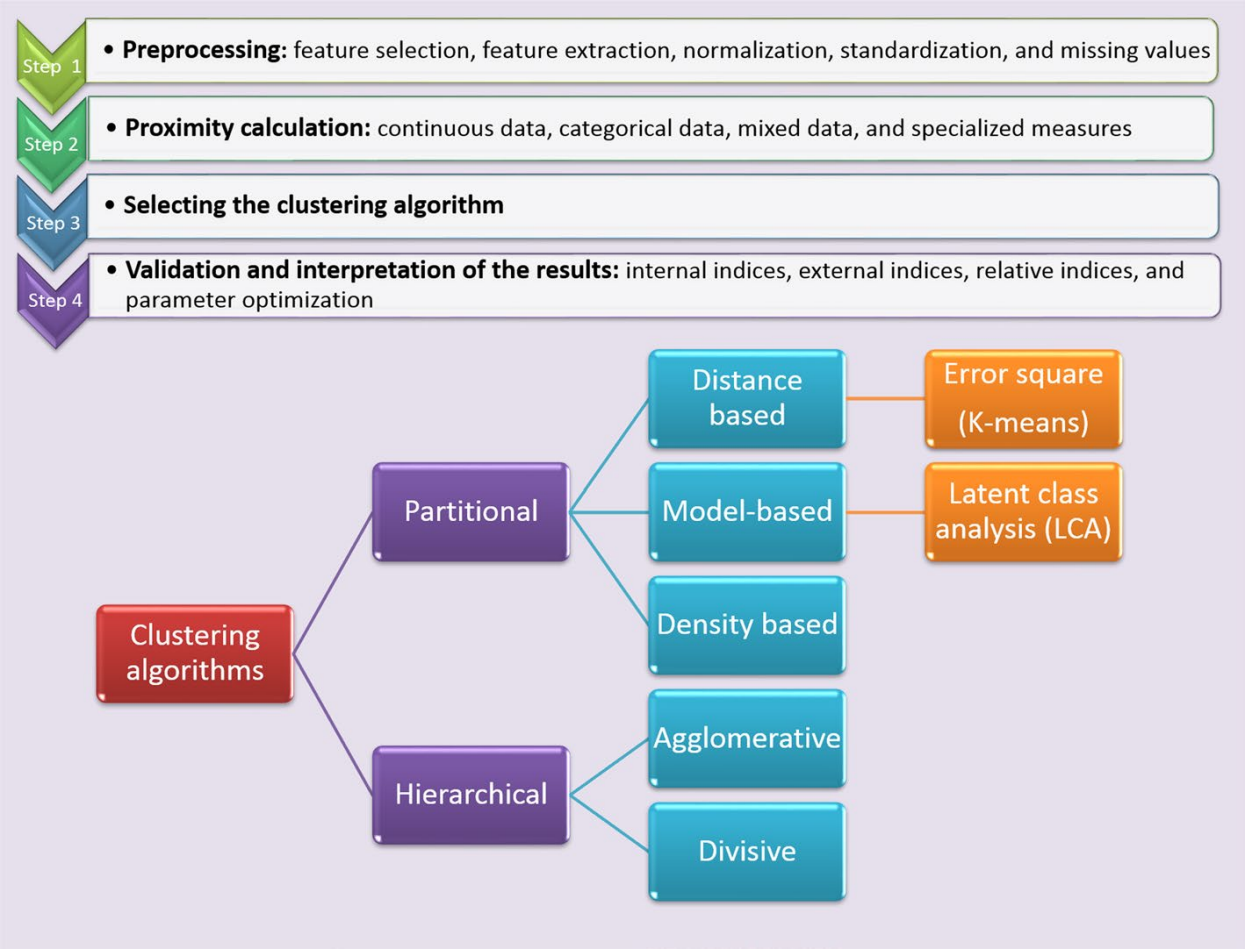
Clustering algorithms

CA is a group of machine learning algorithms that classify data into homogenous groups with the least similarity to other groups [32]. There are different types of clustering algorithms (Fig. 1). K-means CA and hierarchical cluster analysis (HCA) are the most frequently used [33]. K-means CA is preferable due to its good measurement capability. One of the features of this algorithm is the need to calculate the number of clusters before analysis under the title of K [34]. There are different methods for choosing optimal cluster numbers, for example, BIC, AIC, elbow, etc., in K-means CA. The distance metric is another important feature in K-means CA, which uses Euclidian.

HCA converts a distance matrix of all items’ similarity measurements into a hierarchy of nested groups. In this method, two different approaches are used: agglomerative and divisive [34]. HCA is aiming to group similar objects together based on their attributes and characteristics. It involves constructing a hierarchy of clusters, where each object begins as a separate cluster and is progressively merged with others to create larger clusters. This process continues until all subjects are consolidated into a single cluster or until a predetermined stopping condition is satisfied [14]. Latent class analysis (LCA) is another popular method that is a kind of finite mixture model (FMM). In this method, hidden clusters are uncovered by some predetermined multifactorial feature [35]. LCA estimated the probability of belonging to each latent class for each individual allowing researchers to understand the heterogeneity within a population. By uncovering these latent classes LCA provides insights into the structure and patterns of categorical data [36]. Principal component analysis (PCA) is a method that decreases multi-dimensional data before analysis [37], increases interoperability of the results, and minimizes information bias. PCA does the analysis by using new uncorrelated variables [38].

#### Cluster validation

One of the most critical steps in CA is the evaluation of the clusters obtained from the analysis. There are some methods for this assessment, such as Silhouette and Davies-Bouldin indexes [28, 39].

#### Interpretation of the results

The main goal in conducting CA studies is to obtain subgroups and relevant individual characteristics. CA is insufficient in determining the characteristics of clusters and assessing the relationship between different variables. So, after the analysis results are prepared, other methods apply to interpret the results, for instance, using Bayesian inference.

## Results

**Fig. 2:**
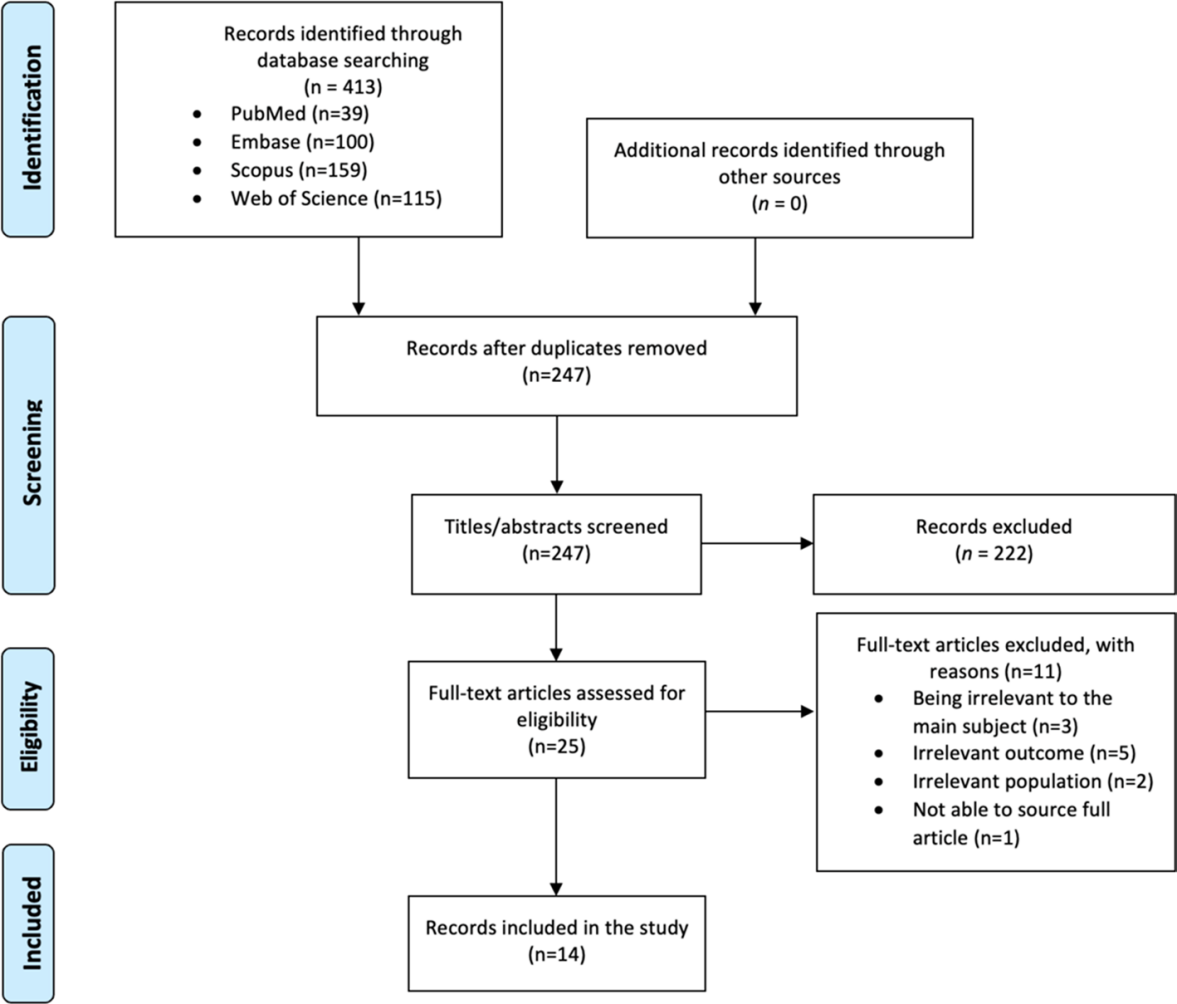
Study selection

As illustrated in Fig. 2, the database search retrieved 413 records. One hundred sixty-six records were duplicated. We screened 247 discrete records by title and abstract, of which 25 appeared potentially eligible. During full-text reviewing, we excluded 11 articles due to not assessing outcomes of interest [40–44], not using CA [45–47], not including IBS patients [48, 49], and not available full-text [50]. Finally, 14 eligible articles were included in this article. The included articles were published between 1995 and 2021.

### Study design

Eight studies were designed as prospective cohort studies [51–58]. Seven of them recruited two groups of participants [51, 52, 54–57], however, one of them recruited only one group of participants [53]. The two groups included one group of IBS patients and one group of healthy controls, except for [52], which recruited two independent groups of IBS patients. Five studies were cross-sectional [42, 59–62]. Among them, two studies had a group of IBS patients [42, 61]. Other studies included two groups of IBS patients [59, 60], except for [62], which recruited one IBS group and one healthy control group. Only one of the studies conducted in 2013 was a randomized clinical trial (RCT) [63], which included patients and healthy controls.

### Sample characteristics

Studies mostly included at least 100 participants [42, 51, 52, 55, 57, 59, 60, 62], except for six [53, 54, 56, 58, 61, 63]. The number of participants varied from 52 to 1533 across studies. Three studies did not report the percentage of participants by gender [53, 55, 56]. In six of the other 11 studies, more than 80% of the participants were female [51, 54, 59–62]. Participants’ age ranged from 17 [52] to 88 [57] years.

### Diagnostic criteria

The included studies were conducted in different years and used different criteria for diagnosis. Three of the initial studies used the ROME I criteria [51–53, 57], and the next five studies that started in 2006 used the ROME II version [54, 55, 58, 61, 62]. Two studies used only the ROME III version [56, 63], and two of the most recent studies used two different criteria to identify IBS patients [59]. The most recent study conducted in 2021 by Black et al*.* used both ROME III and ROME IV criteria in order to identify IBS patients. Han et al. used ROME II and ROME III criteria. One of the studies made the diagnosis based only on the opinion of doctors without any use of questionnaires [42].

### Variables

Table 1 shows that the included studies investigated a wide range of variables. Briefly, seven of these studies clustered patients based on clinical symptoms, QoL, etc. [42, 51, 52, 59–61, 63]. Four of the studies investigated the anocolorectal function of patients and clustering based on it [53–55, 57]. Two studies evaluated immunological factors such as the level of serum cytokines and the role of MC in the pathophysiology of the disease [56, 62]. Finally, a study was conducted on the intestinal microbial composition of IBS patients [58].

**Table 1:** Characteristics of IBS cluster analysis articles

Study, year [reference]	Study design	Population	Diagnostic criteria	Variables	Data collection
Black et al. 2021 [59]	Cross-sectional (two groups of patients)	1375 IBS patients: mean age, 49.2 (18–86); 1157 females (84.1%); 1080 met ROME III; 811 met ROME IV	ROME III or ROME IV	Gastrointestinal (GI) symptoms, mood, extraintestinal symptoms, GI symptom-specific anxiety, stress	IBS-SSS, PHQ-12, HADS-A, HADS-D, CPSS, VSI
Han et al. 2019 [60]	Cross-sectional (two groups)	332 IBS patients aged 18–70 (283 females, 85.2%), 224 patients in group 1 mean age 43.4 (193 females, 86.2%) 108 patients in group 2 mean age 39.7 (90 females, 83.3%	ROME II in group 1 ROME III in group 2	Daily diary of GI and non-GI symptoms, cognitive beliefs, QoL	26-item daily diary, CSFBD, 42-item IBS QoL scale
Lackner et al. 2013 [61]	Cross-sectional (1 group)	98 IBS patients mean-aged 46.7 (female 88%)	ROME II	GI symptoms, QoL	IBS-SSS, UCLA-SSS
Nevé et al. 2013 [63]	Randomized Clinical Trial (RCT)	43 IBS patients aged 18–71(35 female), 29 healthy controls aged 22–62 (20 female)	ROME III	GI symptoms, psychological symptoms, exhaled H2 and CH4	GSRS, HAD, QuinTron Breath Tracker (measure exhaled H2 and CH4)
Eslick et al. 2004 [42]	Cross-sectional	897 patients: mean aged, 44; 577 females (64%); 212 IBS patients (24.5%)	Diagnose by the physicians	GI symptoms	BDQ
Guthrie et al. 2003 [51]	Prospective cohort study (2 groups)	107 IBS patients aged 18–65 (88 females, 82%) and 23 healthy controls aged 20–45 (17 females)	ROME I	Bowel symptoms, psychological assessments, QoL, physiological assessments	BDQ, 11-items Lickert Scale, SCAN, SCL-90-R, a validated screening questionnaire developed by Leserman and colleagues, IIP-32, SF-36, well-lubricated tube for sensory response to rectal distention
Ragnarsson et al. 1999 [52]	Prospective cohort (2 groups)	2 samples Sample 1: 63 IBS patients aged 17–74 (40 females,23 male) Sample 2: 52 IBS patients aged 19–76 (39 females, 13 males)	ROME I	Bowel habits, pain, and, bloating	Daily records of their symptoms for 6 weeks in sample 1 and a week in sample 2
Howard Mertz et al. 1995 [57]	Prospective cohort study (2 groups)	100 IBS patients mean aged 44 (22–88), 69 females 15 healthy controls mean aged 37 (28–50), 7 females	ROME criteria	Anorectal sensory, motor function, GI, and psychological symptoms	The volume-displacement device, anorectal manometry, BDQ, SCL-90
Ragnarsson et al. 1999 [53]	Prospective cohort study	52 IBS patients aged 19–76	Confirmed the diagnosis by gastroenterologist and ROME criteria	Anal sphincter function, anal canal motility, the sensory function of the rectum, RAIR, rectal reservoir function, and rectal motility. GI symptoms, pain, and distention	1-week diary record card, pre and postprandial anorectal manovolumetry
Bouchoucha et al. 1999 [54]	Prospective cohort Study (2 groups)	60 IBS patients aged 21–72 (4 males, 7%) 20 healthy control females aged 22–65	ROME criteria	Anal pressure waves in rest and distention state	Standard manometry
Bouchoucha et al. 2006 [55]	Prospective cohort study (2 groups)	(148 healthy controls and 1385 IBS patients)	ROME II	Colon Transit Time (CTT)	CTT by Prodimed Le Plessis-Bouchard
Bennet et al. 2018 [62]	Cross-sectional (2 groups)	246 IBS patients mean-aged 33 (25–45) (77.2% female), 21 healthy control mean-aged 30 (26.5–43.5) (100% female)	ROME II	Systemic cytokines level, colonic sensory and motility testing, individual assessment questionnaire	High sensitivity multiplex assays, a combined mano-reentry and a barostat, IBS-SSS, IBS QoL, comorbid conditions, brief symptom inventory
Johanna Sundin et al. 2019 [56]	Prospective cohort (2 groups)	43 IBS patients aged 25–44, 20 healthy controls aged 24–38	ROME III	Visceral sensitivity, IgE levels, IBS symptoms, mood, mast cell microscopy and quantification, fecal protease activity assay, mucosal gene expression analysis	Immunoassay, dual drive barostat, IBS_SSS, GSRS-IBS, HADS, VSI, immuno-fluorescence, quantitative reverse transcription PCR
Ian et al. 2014 [58]	Prospective cohort study (2 groups)	37 patients: mean age, 37; 26 females 20 healthy controls (matched age and gender)	ROME II	Microbial composition, GI and psychological symptoms, QoL, rectal sensitivity test, and, CTT	Bioinformatics processes, HADS, GSRS-IBS, SF_36, BSFS, rectal barostat, radio-opaque markers

*IBS-SSS* irritable bowel syndrome severity scale score, *PHQ-12* patient health questionnaire-12, *HADS-A* hospital anxiety and depression scale- anxiety, *HADS-D* hospital anxiety and depression scale-depression, *CPSS* cohen perceived stress scale, *VSI* visceral sensitivity index, *GSRS* gastrointestinal symptoms rating scale, *UCLA-SSS* global severity of GI symptoms scale, *CSFBD* cognitive scale for functional bowel disorders, *SCAN* schedules for clinical assessment in neuropsychiatry, *SCL-90-R* symptom check-list-90-R, *IIP-32* inventory of interpersonal problems-32 items, *SF-36* the 36 item short form survey, *BDQ* bowel disease questionnaire, *RAIR* recto anal inhibitory reflex

### Data collection

Various questionnaires were used to collect the necessary data in the field of clinical symptoms, as shown in Table 1. Specialized tools were used to collect other data; for instance, QuinTron Breath Tracker for evaluating exhaled H2 and CH4 [63], manometry for anocolorectal function, Prodimed Le Plessis-Bouchard for CTT, high-sensitivity multiplex assays [62], immunofluorescence, and immunoassays for systemic cytokines and MC characteristics, RT-PCR for gene expression [56], also bioinformatics for microbial composition.

### Data preprocessing

Four of the studies did not mention details in this regard [51, 56, 62, 64]. Ragnarsson et al. transformed data to have a mean of 0 and a standard deviation of 1 [52, 53]. Exploratory factor analysis was used in two of the studies [60, 61]. Two other studies normalized data in different ways. Bouchoucha et al. [54] normalized data by subtracting the pressure of the first measured point from the measured values in each experiment; however, Jeffery et al. [58] normalized data by scaling to an intensity of 1 to control for differing numbers of reads. PCA and factor analysis were used in two studies [42, 63]. Mertz et al. [57] standardized data and used unpaired student t-tests. One study used a t-test and a partially overlapping z-test [59].

### Determination of cluster numbers

In four of the studies that used the K-mean CA for clustering, the number of clusters was determined based on Euclidian distance [42, 51–53]. Two other studies that used K-mean CA for clustering used pseudo-f statistic [55, 57]. Another study used successive solutions that increment the value of k by 1 [61]. Likelihood-based methods were used in two of the articles that were clustered by using LCA. Han et al. [60] used likelihood‐based criteria and model entropy, and Black et al. [59] used the Bayesian information criterion of the log-likelihood (BIC(LL)) to identify the number of clusters. Natural breaks in distance jumps were used before HCA in one of the studies [63]. The remaining four studies did not mention the use of any methods to determine the number of clusters before clustering [54, 56, 62, 63] (Table 2).

**Table 2:** Methodology of IBS cluster analysis articles

Study, year [reference]	Preprocessing methods	Determination of clusters number	Clustering algorithms	Validation	Interpretation of results
Black et al. 2021 [59]	T-test and partially overlapping z-test	Bayesian information criterion of the log-likelihood (BIC(LL))	Latent class analysis	tenfold cross-validation	χ2 test, ANOVA
Han et al. 2019 [60]	EFA using the principal component method	likelihood‐based criteria and model entropy	Latent class analysis	NR	Χ^2^ test, ANOVA R2 and Cohen’s d
Lackner et al. 2013 [61]	Simple linear transformation, CFA based on maximum likelihood methods, EFA based on principal components extraction	Successive solutions that increment the value of k by 1	K-means cluster analysis	Exploratory cluster analysis	Squared semi-partial correlation
Nevé et al. 2013 [63]	PFA	A natural break in distance jumps	Ascending hierarchical cluster analysis	Silhouette coefficient	ANOVA, correlation matrix, and scatterplots
Eslick et al. 2004 [42]	Factor analysis, PCA, varimax rotation	Euclidian distance	K-means cluster analysis	NR	Describing a cluster profile that comprised the mean score per factor per cluster and, no cluster could be made up of less than 5% of the entire sample
Guthrie et al. 2003 [51]	NR	Euclidian distance	K-means cluster analysis	Bonferroni corrected pairwise comparisons	ANOVA or × 2 tests, Pearson correlation coefficients
Ragnarsson et al. 1999 [52]	Transformed, to have a mean of 0 and a standard deviation of 1	Euclidian distance	K-means cluster analysis	NR	Mann–Whitney and Kruskal– Wallis tests, ANOVA
Howard Mertz et al. 1995 [57]	unpaired Student's t-test, standardized	Pseudo f statistic	K-means cluster analysis	NR	Pearson r correlation coefficient, χ^2^ analysis
Ragnarsson et al. 1999 [53]	Transformed, to have a mean of 0 and standard deviation of 1	Squared Euclidian distance	K-means cluster analysis	NR	Mann–Whitney, Kruskal–Wallis tests, ANOVA, Wilcoxon signed-rank test
Bouchoucha et al. 1999 [54]	Normalized by subtracting the pressure of the first measured point from the measured values in each experiment	NR	K-mean cluster analysis	NR	Mann–Whitney test, Wilcoxon signed rank test., correlation coefficient used spearman r
Bouchoucha et al. 2006 [55]	NR	Pseudo-f statistic	K-means cluster analysis	NR	Chi-square test, GLM, ANOVA
Bennet et al. 2018 [62]	NR	NR	Hierarchical cluster analysis	*Q*2	Mann–Whitney *u* test, Kruskal Wallis followed by Dunn’s test, hoteling’s *T*2, OPLS-DA, PCA, Non-parametric spearman’s rank coefficient
Johanna Sundin et al. 2019 [56]	NR	NR	Hierarchical cluster analysis	Cross-validation by *Q*2 parameter	OPLS‐ DA, Mann‐Whitney, and Kruskal‐Wallis followed by Dunn's test, Hoteling’s *T*2
Ian et al. 2014 [58]	Normalized by scaling to an intensity of 1 to control for differing numbers of reads	NR	Unsupervised hierarchical cluster analysis	NR	Pearson correlation coefficient test, Kruskal Wallis and Mann Whitney test, Blast method, PMANOVA

*EFA* exploratory factor analysis, *GLM* generalized linear model, *OPLS-DA* orthogonal partial least squares-discriminant analysis, *ANOVA* one-way analysis of variance, *CFA* confirmatory factor analysis, *PFA* principle factor analysis, *PCA* principle component analysis

### Clustering algorithms

K-means CA is the most commonly used algorithm. Eight articles used the K-means CA algorithm for clustering [42, 51–55, 57, 61]; four studies used HCA [56, 58, 62, 63]; only two studies used the LCA algorithm for clustering [59, 60] (Table 2).

### Cluster validation

Six studies used methods for cluster validation. Two studies used cross-validation methods. Black et al. [59] used tenfold cross-validation and Sundin et al. [56] used cross-validation by the *Q*2 parameter. Bennet et al. [62] used Q2. Three other validation methods were exploratory CA [61], silhouette coefficient [63], and Bonferroni corrected pair-wise comparisons [51].

#### Interpretation of results

The number of clusters varied from two to seven based on different factors. As shown in Table 2, different methods were used to interpret the results, and find the relationship between different factors. The most prevalent methods were used is as follows: one-way analysis of variance (ANOVA) [51, 53–55, 59, 60, 63], Kruskal Wallis test [52, 53, 56, 60, 62], Mann–Whitney test [52–54, 56, 58, 62], and χ2 test [51, 57, 59, 60]. Other methods include squared semi-partial correlation [61], spearman correlation coefficient [54, 62], and Pearson correlation coefficient [51, 57, 58]. Eslick et al*.* [42] described a cluster profile that comprised the mean score per factor per cluster.

#### Clusters’ profile

Seven articles were based on clinical findings [42, 51, 53, 59–61, 63], four articles were based on anocolorectal functions [52, 54, 55, 57], two studies assessed immunological factors in IBS patients [56, 62], and one study was about microbial composition in IBS [58].

#### Clinical features

As Table 3 shows, seven articles that classified patients based on clinical symptoms are different in several ways, including study design, sample size, diagnostic criteria, clustering algorithms, and findings [42, 51, 52, 59–61, 63]. Some of these studies had similar results in terms of the number of clusters and classification of patients into homogenous groups based on clinical symptoms. Two of the earliest studies classified participants into three homogenous groups [52, 53]. They were similar in diagnostic criteria and clustering algorithm and had almost the same sample sizes.

**Table 3:** Number of clusters, characteristics, and associations

Study, year [reference]	Cluster’s profile	Findings
Black et al. 2021 [59]	Seven clusters in the ROME IV cohort group 1. (N = 161) Diarrhea and urgency with the low psychological burden 2. (N = 170) Lower overall GI symptom severity with the high psychological burden 3. (N = 165) Lower GI symptom severity with the low psychological burden 4. (N = 154) Diarrhea, abdominal pain, and urgency with the high psychological burden 5. (N = 31) Constipation, abdominal pain, bloating, with a high psychological burden 6. (N = 71) High several GI symptom severity with the high psychological burden 7. (N = 59) Constipation and bloating with the low psychological burden The result was almost the same in the ROME III cohort group	Males have overall low symptoms and psychologic co-morbidities, people who have higher psychological co-morbidity suffer from a higher score in all psychological health measurements and, stool subtypes correlate with GI symptoms in each cluster
Han et al. 2019 [60]	Four clusters 1. (N = 153) Low symptoms and good QoL 2. (N = 106) Low symptoms and moderate QoL 3. (N = 38) High symptoms with diarrhea and poor QoL 4. (N = 35) High symptoms with low diarrhea and moderate QoL	Associations: gender with cluster’s memberships, college degree and paid employment with lower symptoms and higher QoL, and history of MDD with class four membership. Diarrhea/urgency symptoms are an important component of IBS symptom profiles
Lackner et al. 2013 [61]	Four clusters 1. (25%) High level of bowel dissatisfaction and pain frequency and QoL and low pain severity 2. (19%) Intermediate score of pain frequency and severity but a high score of bowel dissatisfaction 3. (18%) High score on all IBS-SSS 4. (37%) Low score in all dimensions except bowel dissatisfaction and QoL	Associations, total IBS-SSS score correlates with individual items of the IBS-SSS. Global characterization of IBS symptom severity was highest for cluster 3 patients, lowest for cluster 4 patients, and somewhat elevated for cluster 1 and 2 patients
Nevé et al. 2013 [63]	Five clusters grouped in 2 subpopulations 1. High GI symptom group (clusters 2 + 3 + 5 N = 16) 2. Low GI symptom group (clusters 1 + 4 N = 26)	There is no association between exhaled H2 and CH4 and IBS clusters, a test meal containing lactulose can induce IBS symptoms and distinguish IBS from healthy controls
Eslick et al. 2004 [42]	Seven clusters 1. Diarrhea (24.7%) 2. Meal-related pain (12.1%) 3. Abdominal pain (7.9%) 4. Faucal indicators (12.6%) 5. Nausea/vomiting/weight loss (14.4%) 6. Constipation (8.8%) 7. Undifferentiated (19.5%)	Almost half of IBS patients have diarrhea or constipation and the remaining have mixed symptoms The results show we can differentiate patients into upper and lower GI disorders
Guthrie et al. 2003 [51]	Three clusters 1. (N = 15) Low distension thresholds and diarrhea-predominant or alternating 2. (N = 62) Low distention thresholds and diarrhea-predominant or alternating, moderate prevalence of psychiatric disorders, and low rate of childhood sexual abuse 3. (N = 30) Higher distention thresholds and constipation-predominant or alternating, lower rates of psychiatric disorders, and low rates of medical consultations, sexual abuse, and interpersonal difficulties	Rectal sensitivity does not associate with sexual abuse. There is a relationship between IBS patients with low discomfort threshold and, sexual abuse, discomfort threshold, the severity of pain, psychiatric complexity, and treatment seeking Also, there is an association between high consultation and psychiatric disorders
Ragnarsson et al. 1999 [52]	Three subgroups in both samples 1. (N = 31) Hard, varying consistency, and disturbing passage of stool 2. (N = 35) Loose stool and urgency 3. (N = 38) Normal stool and least disturbed passage Two pain/bloating subgroups, one of them by little and the other by considerable pain and bloating. No relation was found between pain/bloating and bowel habit subgroup membership	The degree of pain and bloating does not associate with the type of bowel habit No significant relation was found between sex and IBS subgroups
Howard Mertz et al. 1995 [57]	Three clusters 1. (N = 30) Hypersensitivity to phasic rectal distention, elevated anal pressures in both rest and stimulation phase, younger, diarrhea-predominant 2. (N = 29) Normal rectal sensitivity in phasic distention and hyposensitivity to the ramp, constipation-predominant 3. (N = 32) Hypersensitivity in phasic rectal distention, increased rectal compliance, constipation-predominant, hard stool, and severe symptoms	Longitudinal follow-up shows an association between symptom severity and changes in perception thresholds Altered rectal perception must be a reliable biological marker in IBS
Ragnarsson et al. 1999 [53]	Three clusters were identified by cluster analysis of the pre-prandial manovolumetry 1. (N = 13) Increased anal canal pressure, and large rectal compliance, with more men than women 2. (N = 18) Decreased rectal sensitivity, with predominantly women and the oldest age range 3. (N = 19) Large fluctuation in anal canal motility, the lowest threshold for causing the recto sphincteric-inhibitory reflex, and the least prominent maximal repetitive rectal contraction Three clusters of bowel habits 1. (N = 17) Hard stools and highly disturbed stool passage 2. (N = 18) Loose stool and urgency 3. (N = 17) Normal consistency stool and the least disturbed stool Two clusters of pain, bloating 1. (N = 39) With a low burden of symptoms 2. (N = 13) With a high burden of symptoms	The anorectal function does not associate with symptoms Changes in rectal sensitivity do not correlate with symptoms Rectal sensitivity was higher in women and rectal compliance was higher in men
Bouchoucha et al. 1999 [54]	Three clusters 1. Ultra-slow waves 2. Slow waves 3. Simple waves	Ultraslow waves are associated with high anal pressure, anal hypertonia, and dyschezia. Ultra-slow waves in IBS patients are not significantly different from healthy controls. Increased activity of anal sphincters associated with ultra-slow waves
Bouchoucha et al. 2006 [55]	Four clusters in patients 1. (N = 170 female + 61 male) Delayed colon transit time in the right colon 2. (N = 223 female + 61 male) Delayed colon transit time in the left colon 3. (N = 107 female + 50 male) Delayed in rectosigmoid area 4. (N = 58 female + 21male) With no markers in plain film Three clusters in the control 1. (N = 27 female + 16 male) Delayed in the right colon 2. (N = 14 female + 35 male) Delayed in the left colon 3. (N = 16female + 26male) Delayed in rectosigmoid area	IBS patients are different from healthy control in colon transit time, marker distribution of retention, and diffusion coefficient
Bennet et al. 2018 [62]	Four clusters 1. (N = 7 HS + 18 patients) Having the lowest level of cytokines 2. (N = 11 HS + 105 patients) Moderate level of serum cytokines cluster 3. (N = 2 HS + 72 patients) Moderate level of serum cytokines cluster 4. (N = 0 HS + 49 patients) Having the highest level of serum cytokines	Systemic levels of immune activation cytokines are elevated in IBS patients, however, there is no strong association between immune activation and IBS symptoms. Also, systemic cytokines are not associated with comorbidities in IBS patients
Johanna Sundin et al. 2019 [56]	Two clusters in both IBS and HC 1. (N = 16 IBS + 10HC) With High MC 2. (N = 27 IBS + 10HC) With low MC	There is no association between MC numbers and location and pathophysiology of IBS, IBS symptoms, and subtypes There is no difference between the two clusters of MC groups based on IgE serum level
Ian et al. 2014 [58]	2 clusters 1. (N = 15) A diminished diversity with a median of 44 2. (N = 22) Increased diversity with a median of 53	Microbial composition correlates with IBS and is associated with immunological alteration and low-grade inflammation

*MDD* major depressive disorder, *MC* mast cell

The study by Ragnarsson et al. [52] was completely based on the participants’ statements. There was no significant difference in the number of patients in all three subgroups. They also clustered IBS patients based on pain and bloating. The patients were divided into two groups with almost equal sample size. In the first group, the symptoms were low, whereas, in the second group, the symptoms were high. Guthrie and his colleagues [51] included patients who suffered from a more severe and chronic form of the disease. Most of the patients were fallen into the second group with a low threshold of rectal sensitivity, diarrhea-predominant or intermittent, and low level of mental disorders.

In two studies, four subgroups were emerged. In [60], which is more recent, the sample size was much larger than in [61] and ROME II and ROME III criteria were used to identify patients. More than 75% of patients were classified in the first and second subgroups, with low symptoms and relatively good QoL.

Lackner et al. [61], included patients with moderate to high severity of symptoms. They had to conduct the classification by using LCA due to the small sample size. The highest severity of symptoms was in the third subgroup, which included a smaller number of patients. More than one-third of the patients were labeled as fourth subgroup, with the lowest severity of symptoms and the highest QoL.

Based on the results of two other studies, the patients were divided into seven subgroups. Both of these studies had a significant sample size. In a study conducted in 2004 [42], a quarter of the patients were classified in the diarrhea-predominant group and about 20% of the patients were classified in the undifferentiated group. In [59], the most novel study in this field, all stages of diagnosis of patients and the data collection were online. The second subgroup included the largest number of patients among the subgroups, in which GI symptoms were low, whereas psychological disturbances were high. There was a significant difference between different clusters in terms of age and gender. For instance, cluster one included mostly elderly patients, cluster five included younger patients, and cluster three included more men.

In 2012, an RCT was conducted [63] to assess the occurrence of symptoms after meals. They evaluated GI symptoms, and psychological symptoms and exhaled H2 and CH4 after lactulose diets. They used three types of meals including 4oo ml liquid plus three different doses of lactulose (0–15 g–25 g). GI symptoms and discomfort were assessed at baseline and every 15 min after different test meals. Both lactulose-containing diets increased GI symptoms in IBS patients; however, the lactulose (25 gr) diet discriminated patients from controls more precisely. Ascending CA (wards method) was performed based on the response after a lactulose test meal and consequently, five clusters were obtained which could be divided into two subpopulations, labeled as, high GI symptoms and low GI symptoms. In the high GI subpopulation, both hospital anxiety and depression scale (HADS) and visceral sensitivity index (VSI) were high. As a result, no significant difference was found between GI symptoms, IBS clusters, subpopulation, and exhaled H2, and CH4 in any dose of lactulose.

#### Anocolorectal function

We reviewed four articles that evaluated the association between IBS and anorectal dysfunction [53–55, 57]. They are the same in study design, diagnostic criteria, and clustering algorithm which was K-means CA. These studies consistently identified subgroups based on anorectal function and associated factors. Mertz et al. [57] proved that altered rectal perception is a biological marker of IBS. They evaluated anal perception thresholds and reevaluated 15 patients after three months to identify the correlation between changes in perception thresholds and symptom severity. Finally, they found three subgroups of IBS patients based on eight physiological parameters. Ragnarsson et al. [53] assessed the hypothesis that abdominal symptoms are related to anorectal function in IBS patients. They classified patients based on anorectal functions, bowel habits, pain, and distention. They investigated anorectal function by using manovolumetry before and 40 min after a fatty meal.

K-means CA resulted in three clusters in anorectal functions. It is similar in both pre- and postprandial manovolumetry, except that in postprandial, the first group does not have increased anal canal pressure and has large rectal compliance with no more men than women. In pre-prandial manovolumetry, the third group is more prevalent; however, in postprandial manovolumetry, the second group is more prevalent. Consequently, rectal sensitivity increased after a fatty meal in somehow half of the patients and women are more sensitive. Ragnarsson et al. [52] have also done clustering based on bowel habits, pain, and distention like their previous study which was mentioned earlier in the clinical findings category with the same results. There was no difference between before and after fatty-meal manovolumetry. So, it might be possible to assume that there is no relation between abdominal symptoms and anorectal functions in IBS.

Bouchoucha et al. have done two studies in the field of anorectal function. One study was about anal pressure waves [57] and the other one was CTT [59]. Participants with delays in manometry or CTT were excluded from the studies. It has been caused to eliminate constipation-predominant patients and is a manifestation of selection bias. In the first study [57], manometry has been done by a small balloon tube in two states of rest and distention. Three clusters of anal pressure waves have resulted from K-means CA in both rest and distention states. In the distention state, ultra-slow waves increase in both groups; however, slow waves increase merely in IBS patients, and simple waves decrease in control groups. Ultra-slow waves in IBS patients are not significantly different neither at rest nor in the distention state. These waves are just a manifestation of increased activity of the internal sphincter [38, 39]. In the second study, the association between CTT and IBS was investigated. They used a previously identified technique using radio-opaque markers [40–42]. Three parameters were assessed, including CTT, distribution of markers, and diffusion kinetics. To do this, three different parts of the colon, including the right colon, the left colon, and the rectosigmoid area, were assessed. They identified four clusters in IBS patients and three clusters in healthy controls. In both groups, cluster one has a delay in the right colon, cluster two has a delay in the left colon, and cluster three has a delay in the rectosigmoid area. In the patient’s group cluster four is defined as no marker seen in the plain film. In the IBS group, a higher percentage of females is in cluster two and the number of males was higher equally in both clusters one and two; however, in healthy controls, more females were in cluster two and more males were in cluster one. Total CTT was more in the second cluster in both sexes. Generally, IBS patients have longer CTT than controls, and females have longer CTT. No correlation was found between CTT and IBS.

#### Immunological findings

Two of the studies assessed the correlation between IBS and aggregation of colonic MCs and the level of serum cytokines. The results were diverse, with some studies identifying subgroups based on MC characteristics and cytokine levels.

Sundin et al. [38] intended to discover the pathophysiology of IBS. They determined the mucosal MC characteristics and their proximity to nerves, fecal serine protease activity, symptoms, visceral sensitivity, and expression of epithelial barrier genes. They measured the MC using a method previously published [39]. They analyzed data by HCA and identified two subgroups. One subgroup, MC High, has higher rates of MC and proximity to nerves, and, the second, MC Low, which is the opposite of the previous subgroup. A higher percentage of patients was classified in MC Low subgroup. Different subgroups of IBS could not be distinguished based on symptoms, visceral sensitivity, gene expression, and fecal protease activity. MC numbers and location sound to not have any role in the pathophysiology of IBS.

The role of cytokines and their correlation with IBS was investigated by Bennet et al. [40], who assessed pro-inflammatory factors, including IL1B, IL8, IL6, TNF, and IL10 [41, 42]. They obtained four clusters. Most of the patients and controls were in the second cluster. The first cluster had the lowest level of cytokines, and the fourth cluster had the highest level of cytokines. In all of the clusters, TNF had the highest level in comparison with other cytokines except in the second cluster, in which, IL8 had the highest level. Finally, they identified IBS patients have a higher level of cytokines in comparison with healthy controls. However, this issue cannot differentiate patients from healthy controls and there is no correlation between cytokine levels and IBS symptoms.

#### Microbiome composition

There was one study about microbiome composition in IBS patients [58]. This study found IBS patients have lower microbial diversity compared with healthy controls. They analyzed data by HCA. The results showed less than half of the patients were like normal controls, and the others were classified into two subgroups. The first was characterized by a diminished diversity and a median of 44 species and the second by increased diversity and a median of 53 species. There were some associations between microbial composition and clinical or physiological features, immunological alterations, and low-grade inflammation in IBS patients.

The CA.

## Discussion

Different approaches exist for addressing the heterogeneity grouping problem, including CA, latent class analysis (LCA), and mixture modeling. CA is advantageous in identifying distinct subgroups and providing visual representations but lacks standardized methods and may overlook latent factors [14]. LCA incorporates latent factors and allows for hypothesis testing but assumes conditional independence and requires large sample sizes [65]. Mixture modeling offers flexibility in distributional assumptions and handles missing data but requires complex model estimation and interpretation [66]. The choice of approach depends on the specific research question, data characteristics, and the balance between interpretability and flexibility needed in the analysis. CA is a group of unique machine learning algorithms that identifies different homogenous subgroups in datasets. Due to their unique features, these algorithms are now increasingly used in studies for various purposes [58]. In this review, we included 14 articles that used CA in IBS patients and obtained different subgroups of IBS patients based on different factors. The number of clusters obtained from these studies varied from two to seven. Seven studies were based on clinical symptoms in IBS patients. They selected different variables. The details of clusters are summarized in Table 3. K-means CA was used in four of these studies. In four of these studies, unlike in the past, the classification of patients was based on the severity of symptoms rather than the form of bowel habits, and most of the participants were in clusters with low GI symptoms severity and good QoL. Some specific findings and associations were obtained from these studies (Table 3). Men have lower symptoms in comparison with women, and GI symptoms are based on stool subtypes [59]. However, Ragnarsson et al*.* [52] found completely contradictory results. They identified that there is no association between sex and IBS subgroups and mentioned the degree of pain and bloating does not correlate with the type of bowel habits. Meals can induce symptoms in IBS patients; however, there is no significant association between meals and IBS clusters.

Four studies evaluated anocolorectal function in IBS patients. They used K-means CA. None of them measured the validity of the analysis results. The study conducted by Ragnarsson et al. [53] sub-grouped patients based on rectal manometry results. In addition to this, they subgrouped patients based on bowel habits, pain, and, bloating. They identified three clusters based on manovolumetry, three clusters of bowel habits, and two clusters of pain and bloating. The main results of this study include: the anorectal function does not correlate with symptoms; changes in rectal sensitivity do not associate with symptoms; rectal sensitivity is higher in women; and rectal compliance is higher in men.

Bouchoucha et al. [54] investigated different types of anal pressure waves in IBS patients. Three clusters were obtained, including ultra-slow waves, slow waves, and simple waves. They found that ultra-slow waves associate with high anal pressure, anal hypertonia, and, dyschezia. Ultra-slow waves in IBS patients are not significantly different from healthy controls. Increased activity of anal sphincters is associated with ultra-slow waves. Altered rectal perception is a biological marker of IBS and its change is associated with symptom severity [57]. IBS patients are different from healthy controls in CTT, marker distribution of retention, and diffusion coefficient. Most IBS patients have delayed CTT in the left colon [55]. We reviewed two studies in the field of immunological findings of IBS patients that utilized HCA. They evaluated systemic cytokine levels, MC number, and location in intestinal epithelium to identify its role in IBS pathophysiology. They found no strong associations between immune activation and IBS symptoms. MC numbers and location do not correlate with the pathophysiology of IBS, symptoms, and subtypes. A study evaluated the gut microbial composition of patients and found that microbial composition correlates with IBS and is associated with immunological alteration and low-grade inflammation.

These studies resulted in inconsistent findings and they are not comparable. They are different in sample size, diagnostic criteria, methodology, etc.

The CA conducted in the studies included in this systematic review, demonstrated moderate consistency with clinical criteria in several aspects of irritable bowel syndrome (IBS). These subgroups were consistent with certain clinical criteria such as symptom severity, bowel habits, pain, and physiological parameters. However, there were also instances where cluster analysis did not show a strong correlation with clinical criteria, indicating the complexity and heterogeneity of IBS.

In terms of clinical symptoms clustering, some studies found consistent results, indicating the presence of homogenous patient groups. However, the findings related to anorectal function clustering were consistently supportive, suggesting the potential for tailored treatments based on symptom profiles and associated factors. On the other hand, the immune feature clustering studies yielded inconsistent results, highlighting the need for further exploration and validation. Given the variability in findings, combining the most clinically consistent variables may improve patient stratification and guide personalized treatment approaches. However, it is essential to thoroughly assess the reliability, validity, and generalizability of each variable before their combination.

Some limitations of these studies are summarized in Table 4. The major limitation was the small sample size. The sample size is a crucial factor in studies and as larger the sample size is, the results can be more generalizable. Also, some of the studies had selection bias in some way. For instance, most of the participants were related to one center and were in the severe spectrum of the disease, so the results cannot be generalized to the whole spectrum of patients. Most of the published studies applicated subjective data.

**Table 4:** Limitations and future suggestions

Study, year [reference]	Limitations	Suggestions
Black et al. 2021 [59]	The diagnosis 0f IBS did not confirm, the questionnaire was online and it is unclear who responded to them or are those who answered suffering from IBS and whether their answers could be generalizable, since internet access is required, many could not participate and some people may have left the questionnaire in the middle. And finally, factors that affected the quality of life did not evaluate	Future studies need to predict underlying pathophysiological mechanisms in each cluster and, their specific treatments and, collect future treatment trials to achieve personal treatment
Han et al. [60]	Most of the participants were middle-aged Caucasian women so, it cannot be generalizable, and the cause-and-effect relationship between the characteristics of the patient and clusters could not be investigated	Future research should do the study based on ROME IV criteria, and asses the correlation between biomarkers and latent classes to target therapy
Lackner et al. 2013 [61]	The sample size is small and limited to just one clinic, there are some biases because of self-reported data, and k-means classification is not optimal however, there is no other choice because of the small sample size and also the symptoms intermittency could not assess	Future research should assess the biobehavioral variables and use instruments to define a subgroup scheme, and investigate the temporality of symptoms by longitudinal studies
Nevé et al. 2013 [63]	It cannot be generalized to all patients because of the small sample size and collect a sample from a tertiary center that has more severe IBS	Future research should be done in a larger population, analyze gut microbiota composition and compare the lactulose challenge test with a standard visceral sensitivity
Eslick et al. 2004 [42]	This questionnaire does not measure all of the diagnostic criteria, referral bias may be present because this physician has more IBS patients due to his specialty, and this study couldn’t compare the patients with other countries and show the temporality of symptoms	Future research should be larger and include more physician practices, including participants from different countries to investigate the effect of different cultures on functional gastrointestinal disorders
Guthrie et al. 2003 [51]	40% of patients refused rectal sensitivity measurements, this study cannot be generalized to all IBS patients because, it was conducted in secondary and tertiary centers and, in a severe range of patients	Future research must identify specific subgroups to develop target therapy
Ragnarsson et al. 1999 [52]	Medication use in sample 1 might influence the results	Future research should conduct to identify the pathophysiological mechanism of these subgroups and consequently target therapy in different patients
Howard Mertz et al. [57]	The sample size was small and reported symptom changes may have been affected by memory bias	Future studies should replicate these subgroups in other IBS populations, evaluate symptomatically correlates between patterns of physiologic parameters, and address regional autonomic abnormalities that might manifest by altered intestinal compliance, fluid, and electrolyte handling, and mucus secretion
Ragnarsson et al. 1999 [53]	This study could not show the association between pre- and post-prandial anorectal function and gastrointestinal symptoms	Future research should enroll more men to assess the sex difference in different parameters and, this method should repeat to identify the etiology and mechanism of the disease
Bouchoucha et al. 1999 [54]	In the present study, the use of visual detection of ultra-slow waves give little information about possible differences be- tween the two groups of subjects	More studies should be conducted to investigate whether these groups overlap or whether they include different groups of patients. The results of the present study must be compared with those of earlier studies of rectal slow waves
Bouchoucha et al. 2006 [55]	It has a selection bias because the study was conducted in a high-care center and rectal manometry was performed in people with constipation, which caused us to have more patients in this study than the general population	As regards, healthy people are not normally distributed in the current study, in future studies, researchers should be careful about the participants enrolled in the study
Bennet et al. 2018 [62]	The number of controls is very small compared to patients. Due to the nature of the study, it is not possible to determine whether cytokines have a delayed effect on symptoms or if they may have increased due to other reasons	Future research should conduct to identify the source of increased cytokines, cytokine cutoffs to identify immune-activated patients, the pathophysiology of immune activation in IBS, and, the way that triggers symptoms
Johanna Sundinet al. 2019 [56]	It is not able to identify the difference in the amount of mast cells and their proximity to the nerves in the large intestine mucosa of patients and controls	Future research should evaluate geographical differences in the pathophysiology of IBS by using standardized biopsy sampling, standardized inclusion, and exclusion criteria, and conducted in different geographical locations
Ian et al. 2014 [58]	It could not control probiotic consumption, which might theoretically cause confounding effects on microbiota composition	Future research should consider some antibiotic-free periods before sampling, and investigate microbial composition role in IBS

The fact that emerged from this review is that IBS is not merely a GI disorder, but it is a disease that affects many things. The most important effect is a psychological disturbance. Also, changes in the diversity of the intestinal microbiome, such as an increase of Firmicutes-associated taxa and depletion of Bacteroidetes-related taxa, and also aberrations in cytokines can be underlying mechanisms. In summary, CA is a type of unsupervised learning technique that eliminates the need for experts to spend time on manual labeling, making it a convenient method. Nevertheless, it is crucial to acknowledge that CA is greatly influenced by hyperparameters, including the number of clusters and the random initialization of cluster centers. The arbitrary selection of these parameters can result in different outcomes, even when employing the same algorithm. The utilization of CA has provided valuable insights into the heterogeneity of IBS based on clinical features, anocolorectal functions, immunological factors, and microbiome composition. Although there were variations in the clustering results, some consistent patterns emerged. However, no particular clustering method or k-means cluster number method consistently outperforms others in terms of consistency with clinical criteria. Further research is needed to explore the optimal clustering approach for accurately capturing the clinical heterogeneity in IBS. Other suggestions for IBS clustering are proposed in Table 4. It is better to have a larger sample size, normally distributed participants in terms of gender and other factors, enroll participants from different geographical locations, and based on ROME IV criteria. As the symptoms are temporary, it is better to conduct studies that follow patients over time to identify the exact pathophysiology of the disease by measuring biomarkers or examining the microbial composition of the intestines, etc., to achieve targeted treatment of the disease. These investigations might be able to reduce additional treatment costs and, the burden of the disease.

## Conclusion

We conclude that unlike the previous classifications, which were based exclusively on bowel habits, CA focuses more on the severity of all symptoms in IBS. Overall, most patients have low severity of clinical symptoms and a good QoL. The clustering based on colorectal function has shown that rectal sensitivity increases in most patients and this can be used as a biological indicator in IBS. The level of serum immunological markers increases moderately in IBS and the diversity of the intestinal microbiome decreases. The number of IBS clusters is variable based on different factors and according to the chosen methodology. As a result, we cannot express a definite number of clusters. Considering that, knowing the different clusters based on different factors would help us to know the disease more accurately, and understand its pathophysiology more precisely, further studies should be done with a similar methodology to be comparable and based on the recommendations mentioned before. So, we hopefully will be able to treat IBS patients in a more targeted manner in the future.

### Supplementary Information

**Additional file 1**. PRISMA 2020 Checklist.

**Additional file 2**. Search strategy.

## Data Availability

All data generated or analyzed during this study are included in this published article.
