# Supplementary material for: Subtyping irritable bowel syndrome using cluster analysis: a systematic review
Source: BMC Bioinformatics. 2023 Dec 15;24:478. doi: 10.1186/s12859-023-05567-8 (PMC10724977; doi:10.1186/s12859-023-05567-8)
Supplement: Supplementary file 2 — Additional file 2. Search strategy. [file 12859_2023_5567_MOESM2_ESM.docx]

Supplemental file 2 Search strategy

PubMed

(((((((((Irritable Bowel Syndromes) OR (Syndrome, Irritable Bowel)) OR (Syndromes, Irritable Bowel)) OR (Colon, Irritable)) OR (Irritable Colon)) OR (Colitis, Mucous)) OR (Colitides, Mucous)) OR (Mucous Colitides)) OR (Mucous Colitis)) AND (((((Analyses, Cluster) OR (Analysis, Cluster)) OR (Cluster Analyses)) OR (Clustering)) OR (Clusterings))

Embase

('irritable bowel syndrome'/exp OR 'irritable bowel syndrome') AND ('cluster analysis'/exp OR 'cluster analysis')

Web of Science

1. TI=(irritable bowel syndrome)
2. AB=(irritable bowel syndrome)
3. AK=(irritable bowel syndrome)
4. #1 OR #2 OR #3
5. TI=(cluster analysis)
6. AB=(cluster analysis)
7. AK=(cluster analysis)
8. #5 OR #6 OR #7
9. #4 AND #8

Scopus

TITLE-ABS-KEY ( irritable  AND bowel  AND syndrome  AND  cluster  AND analysis )
